# Transcriptional Differences between Diapausing and Non-Diapausing *D*. *montana* Females Reared under the Same Photoperiod and Temperature

**DOI:** 10.1371/journal.pone.0161852

**Published:** 2016-08-29

**Authors:** Maaria Kankare, Darren J. Parker, Mikko Merisalo, Tiina S. Salminen, Anneli Hoikkala

**Affiliations:** 1 Department of Biological and Environmental Science, University of Jyväskylä, P.O. Box 35, Jyväskylä, Finland; 2 Centre for Biological Diversity, School of Biology, University of St Andrews, Fife, KY16 9TH, St Andrews, United Kingdom; 3 BioMediTech, Biokatu 6, F1-33014, University of Tampere, Tampere, Finland; University of Arkansas, UNITED STATES

## Abstract

**Background:**

A wide range of insects living at higher latitudes enter diapause at the end of the warm season, which increases their chances of survival through harsh winter conditions. In this study we used RNA sequencing to identify genes involved in adult reproductive diapause in a northern fly species, *Drosophila montana*. Both diapausing and non-diapausing flies were reared under a critical day length and temperature, where about half of the emerging females enter diapause enabling us to eliminate the effects of varying environmental conditions on gene expression patterns of the two types of female flies.

**Results:**

RNA sequencing revealed large differences between gene expression patterns of diapausing and non-diapausing females, especially in genes involved with metabolism, fatty acid biosynthesis, and metal and nucleotide binding. Differently expressed genes included several gene groups, including *myosin*, *actin* and *cytochromeP450* genes, which have been previously associated with diapause. This study also identified new candidate genes, including some involved in cuticular hydrocarbon synthesis or regulation (*desat*1 and *desat2*), and acyl-CoA Δ11-desaturase activity (*CG9747*), and few odorant-binding protein genes (e.g. *Obp44A*). Also, several transposable elements (TEs) showed differential expression between the two female groups motivating future research on their roles in diapause.

**Conclusions:**

Our results demonstrate that the adult reproductive diapause in *D*. *montana* involves changes in the expression level of a variety of genes involved in key processes (e.g. metabolism and fatty acid biosynthesis) which help diapausing females to cope with overwintering. This is consistent with the view that diapause is a complex adaptive phenotype where not only sexual maturation is arrested, but also changes in adult physiology are required in order to survive over the winter.

## Introduction

Seasonally changing environmental conditions pose great challenges for organisms inhabiting northern latitudes, and many species enter some level of dormancy to survive over the cold period. One of the most common types of dormancy in insects is diapause, where growth and/or reproduction are halted over winter and synchronized to resume in a more favorable season [1]. At northern latitudes, seasonal changes in photoperiod are the most reliable signal for forthcoming environmental changes so many insects use this signal to coordinate their diapause [2].

Diapause is considered a dynamic pathway induced by specific environmental cues and it involves various phases [3] and modules including photoperiodism, hormonal events as well as diapause itself [4]. Photoperiodic signals are effective in evoking diapause only during the sensitive period of an insect’s life cycle which is species-specific and may occur well before the actual diapause stage [1]. Seasonal timing of diapause is often defined by measuring the critical photoperiod/day length (CDL): the photoperiod where a diapause response measured by the size of the ovaries is observed in approximately half of the females of a given population, while the other half continues to sexual maturation [5]. CDL is typically a narrow time period because even small changes in day length in either direction can increase or decrease diapause incidence [6]. Consequently, local selection for the optimal timing of diapause at different latitudes can lead to population-specific CDLs and give rise to clines in this trait despite high gene flow between populations [7,8,9]. A longer growing season induced by global warming has already been shown to favour shorter southern CDLs over longer northern ones in several species [10,11].

The sensitive period for diapause induction is followed by a preparative phase during which diapause-destined individuals change their behavioral [12] and feeding patterns [13] in preparation for the adverse season. In adult reproductive diapause, nutrient reserves are accumulated in fat bodies, mainly as lipids (triacylglycerides) [14] but also as glycogen [15] and storage proteins [1,16], at the expense of ovarian development (e.g. [17]). It is critical that diapausing individuals gather enough energy reserves in advance, since feeding is often greatly reduced, if not arrested, during the actual diapause phase, and insufficient reserves can affect both diapause entry and termination [18]. In addition to nutrient reserves, molecular chaperones and cryoprotectants, including heat shock proteins and glycerol, are often synthesized to protect proteins and tissue from stressful conditions such as freezing or desiccation [19,20]. During the actual diapause phase, metabolic activity is suppressed [21] and energy usage is transferred from costly tissues such as flight muscle [22] to more critical systems like the brain [23]. As a result, diapausing individuals are more stress-tolerant and live longer than non-diapausing ones [24,25].

One factor impeding an understanding of insect diapause is variation of the phenotype which may at least be partly because diapause has evolved independently in different insect species [26,27]. Much research on diapause has been directed towards economically important species, such as the silkworm (*B*. *mori*), Colorado potato beetles (*Leptinotarsa decemlineata*), Rice stem borer (*Chilo suppressalis*) and mosquito species that are disease vectors (*e*.*g*. *Aedes aegypti*, *Culex pipiens*), with emphasis on the ecological and physiological aspects of diapause. In contrast, the underlying molecular and genetic causes of diapause are less well known [1,28]. The discovery of adult reproductive diapause in *Drosophila melanogaster* [29] has enabled in-depth research on the genetics of diapause [30–34]. However, the diapause response in *D*. *melanogaster* is shallow, recent in origin, observed only under certain temperatures, and it never reaches a full 100 percent response, i.e. not all females enter into diapause [35,36]. Therefore, wild *Drosophila* species that evolved a robust diapause response in temperate and northern latitudes [37] may be better suited for studies on diapause genetics than *D*. *melanogaster* [1] and bring a new insights and dimensions to these studies.

Here we show gene expression changes linked with adult reproductive diapause at the whole transcriptome level in a northern fly species, *Drosophila montana*, a member of the *Drosophila virilis* group. *D*. *montana* diverged from *D*. *virilis* approximately 9 million years ago [38] and from *D*. *melanogaster* approximately 63 million years ago [39]. *D*. *montana* females overwinter in an adult reproductive diapause, where ovarian development is halted at the pre-vitellogenic stage [40]. The CDL of *D*. *montana* varies along a latitudinal cline and decreases by ca 1.6 h from northern to southern populations in Finland (67–61°N), documenting a strong photoperiodic response and local adaptation even in the presence of high gene flow [9,41]. The flies of this species are also very cold-tolerant [42] and show adaptive changes in their daily and annual locomotor activity rhythms [43]. We performed RNA sequencing (RNAseq; transcriptome) analyses for diapausing (D) and non-diapausing (ND) *D*. *montana* females reared at the same CDL and temperature, where about half of the emerging females will enter diapause, to identify genes that are differentially expressed during reproductive diapause. This experimental design enabled us to eliminate the effects of different environmental conditions on gene expression patterns of D and ND females, a comparison that has been mostly overlooked in previous studies. D and ND females differ dramatically in the relative sizes of their ovaries. Since we used whole-body extractions and this ‘tissue heterogeneity’ could bias our RNAseq results, we performed additional analysis to estimate the extent of bias generated by the size of the ovaries. First, we examined whether the genes that showed differential expression between the two female types in our study were enriched for ovary genes. Second, we conducted qPCR analyses for D and ND females whose ovaries had been removed using a set of genes that had shown differential expression in the RNAseq analysis.

## Material and Methods

### Sample preparation

Our study material consisted of *D*. *montana* females from three isofemale strains (3OL8, 175OJ8 and 265OJ8), each of which was founded from the offspring of a single fertilized female fly collected from Oulanka, Finland (66°N) in 2008. No permission is needed for collecting Drosophila flies in Oulanka (Finland). Since their founding, these strains have been maintained in the laboratory with overlapping generations under diapause preventing conditions (constant light, 19°C and 60% humidity) in plastic bottles containing malt medium [44]. Females of these strains were collected for RNA sequencing in 2010, i.e. after about 16 generations of laboratory maintenance. Diapause response of *D*. *montana* females is genetically determined and does not change under constant light in the laboratory [40].

Virgin, female flies were collected from the malt bottles within one day after eclosion using light CO_2_ anaesthesia and transferred in malt vials into a climate chamber (Sanyo MLR-351H). Flies were reared in this chamber in a light:dark cycle of 18.5:5.5, which represents the critical day length for diapause induction (CDL) for females [9] and corresponds to the beginning of August in the Oulanka population. In our study, ca 10% to 20% of about 50 females dissected in each isofemale strain reared in this CDL showed an intermediate phenotype [9]; all of these females were discarded and we only used D and ND flies that were easiest to identify.

The reproductive state of females was determined by submerging frozen females into RNAlaterICE (Ambion) and dissecting them under a light microscope. The females with pre-vitellogenic, small and transparent ovaries with no yolk accumulation or visible segments were classified as diapausing (D) and the females with large vitellogenic ovaries with visible eggs as non-diapausing (ND) [45]. Females that had intermediate ovaries with some yolk accumulation and visible segments, but no eggs, were discarded. Ten D and ten ND females from each of the 3 strains were pooled to create 3 biological samples of both female types for RNAseq (each strain made up one sample). For the qPCR analysis, we used the RNA extracted from single D and ND females whose ovaries had been removed before RNA extraction. All samples were collected from the environmental chamber at the same time.

### RNA extraction and RNAseq

RNA extraction was performed using a Tri Reagent (Sigma-Aldrich) extraction kit followed by a RNeasy Mini (Qiagen) kit with DNase treatment. RNA concentration and purity was checked using NanoDrop ND-1000 spectrophotometer (NanoDrop Technologies) and its integrity using a 2100 Bioanalyzer (Agilent Technologies). A MicroPoly(A) Purist kit (Ambion AM1919) was used to enrich mRNA from total RNA sample.

Sequencing libraries were constructed from each sample using a SOLiD total RNAseq Kit with unique barcodes (SOLiD Transcriptome Multiplexing Kit) and sequenced in the Finnish Microarray and Sequencing Centre (Turku, Finland) using a SOLiD 5500XL Genetic Analyzer (Applied Biosciences) to generate 75 base pair forward and 35 base pair paired-end reads. Raw sequence reads were trimmed using SOLiD TRIM (with run options: -p 3 -q 22 -y y -e 2 -d 10) to remove polyclonal errors from the data [46] and the reads that passed this filter were corrected using SOLiD Accuracy Enhancer Tools (SAET) to reduce the amount of colour calling errors/erroneous bases in the sequence. Finally, any remaining low quality bases at the end of the reads were trimmed using CLC Genomics Workbench 5.0.1 (CLC Bio http://www.clcbio.com/) (quality score: 0.02).

#### De novo assembly

Since *D*. *montana* does not currently have a reference genome available, a *de novo* assembly of the transcriptome was produced using CLC Bio (available from http://www.clcbio.com/) with default settings. To produce a reference assembly, we used the reads described above, along with the reads produced in two other *D*. *montana* transcriptome projects on the effects of cold acclimation [47] and different photoperiods [48] on gene expression.

#### Annotation of the contigs

Contigs were annotated using Blast2GO [49]. Specifically, all contigs were blasted (blastX) to the non-redundant protein sequence (nr) database. Contigs without a significant blast hit (E-value >0.001) were then blasted (blastN) to non-redundant nucleotide collection (nt). Contigs that still had no hits were blasted (blastN) against the Reference Sequence (RefSeq) genomic database. Contigs that blasted to non-arthropods or rRNA genes were discarded prior to mapping, while contigs that did not get any blast hits were kept in the reference assembly as they could still represent functional genes.

#### Read mapping and gene expression analysis

Reads for each sample were mapped individually to the *de novo* reference assembly using CLCBio. HTSeq [50] was used to quantify the number of uniquely mapping reads to each contig. Differential expression (DE) of contigs between D and ND females was calculated using DESeq package (version 1.9.4, [51] in R (v. 2.14.2; R Foundation for Statistical Computing, Vienna, Austria. https://www.R-project.org/). Variation in library size caused by differences in sequence depths between the samples was taken into account by performing between-library normalization. After this, a generalized linear model (GLM) with a negative binomial distribution was fitted in DESeq with diapause state as a factor. The P values from the GLM were corrected for multiple testing using Benjamini and Hochberg's algorithm [52] to control for false discovery rate (FDR) at P < 0.05.

#### Functional classification

DAVID (v. 6.7) [53] was used to carry out functional clustering for DE contigs at P < 0.0001. Functional categories of the annotation terms over-represented in the study list were ranked based on their level of enrichment defined as the geometric mean of P values for each annotation term within the group [53]. All the contigs with identified orthologs were used as the ‘background’ for comparisons.

#### Estimation of the effect of tissue heterogeneity through enrichment analysis

Substantial differences in the tissue composition of the study samples may cause mis-estimation of gene expression differences between samples [54]. In order to determine whether tissue heterogeneity in the ovary size of D and ND females could explain gene expression differences between females, we examined whether our differentially expressed (DE) genes were enriched for ovary genes. To do this, we used the modENCODE high-throughput RNAseq data from *D*. *melanogaster* [55] to classify *D*. *montana* orthologs as having low, medium, high, or very high ovary expression. We then compared the proportion of these genes in our DE genes (contigs) to the proportion in all genes, using a one-sided Fisher’s exact test implemented in R (R Core Team, 2013).

#### Estimation of the effect of tissue heterogeneity with qPCR

To examine the effects of tissue heterogeneity on the RNAseq data, we also performed qPCR runs with a set of upregulated genes. We performed qPCR runs for D and ND females collected at the same time as the original samples whose ovaries had been removed. Total RNA was extracted from single females without ovaries using a ZR Tissue & Insect RNA MicroPrep Kit with DNAse treatment (Zymo Research), using six females (replicates) from each of the three strains. Purity and integrity of RNA was measured with NanoDrop and TapeStation (Agilent) and cDNA was generated using equal concentrations of RNA (400 ng/μl) and iScript Reverse Transcription Supermix (Bio-Rad Laboratories).

The five genes for the qPCR analyses were selected among new potential candidate genes or on the basis of their earlier detected function in diapause or in cold tolerance (see below), and most of them were picked from the two gene clusters showing the highest number of significantly DE genes in our transcriptome analysis (see results). *Desaturase 1*, *desat1*, and *cyp12a5*, a cytochrome P450 gene were among the largest annotated gene cluster of 285 DE metal ion binding genes. From these, *desat1* has been connected to cold tolerance (e.g. [56]) and *couch potato* (*cpo*), from the nucleotide binding (242 DE genes) cluster, to diapause by Schmidt et al [57] including our earlier studies [58]. *Odorant binding protein 44A*, *Obp44A*, and *period*, *per* were not present in any of the functional clusters identified by DAVID, but were upregulated in diapausing females. Earlier studies have also shown *per* to be upregulated in the initiation, maintenance and overwintering stages in diapausing *D*. *montana* females [59]. We tested several control genes in the samples and *Ribosomal protein L32* (*RpL32*) and *18SrRNA* (*18S*) were selected, as they showed least variation in their expression level in qPCR. Primers for the control and experimental genes were designed using *D*. *montana* sequences and NetPrimer (http://www.premierbiosoft.com/netprimer/). Amplification efficiency of the primers was analysed using 2-fold serial dilutions of pooled cDNA from all the samples (S2 Table). The 20 μl reaction mixes for qPCR contained 10 μl of 2x Power SYBR Green PCR Master Mix (Bio-Rad Laboratories), 0.3 μM of each gene-specific primer and 1 μl of cDNA solution. Cycling conditions in Bio-Rad CFX96 instrument were: 3 min. 95°C, 10 s. 95°C, 10 s. 55°C and 30 s. 72°C (40x), followed by melting curve analysis (65°C-95°C) for amplification specificity checking. Gene expression values for all comparisons were calculated with normalized expression method (ΔΔ^(Ct)^ [60]) using two control genes and real efficiency values. Statistical significance of the expression differences between different comparisons was tested using an ANOVA in R.

## Results

### Transcriptome assembly and gene expression analysis

RNA sequencing produced approximately 37 million paired-end reads of 75 and 35 bases in length. Trimmed reads were assembled into 31880 contigs with a N50 of 527 and the mean contig length of 471 (minimum contig length was 200 bases). We obtained blast results for 99% of the contigs assembled from the RNA sequencing data, and out of them close to 82% blasted to known genes and over 14% to genomic scaffolds in the RefSeq database. As expected, most of the blast hits (>25 000 contigs) were to sequences from *D*. *virilis*, a close relative of *D*. *montana* with a well annotated genome available. Almost all of the remaining hits were to other *Drosophila* species and less than 2% of contigs (647) were discarded as possible contaminants, due to their hit non-arthropod sequences. 321 contigs did not get a significant blast hit, and were retained.

**Fig 1:**
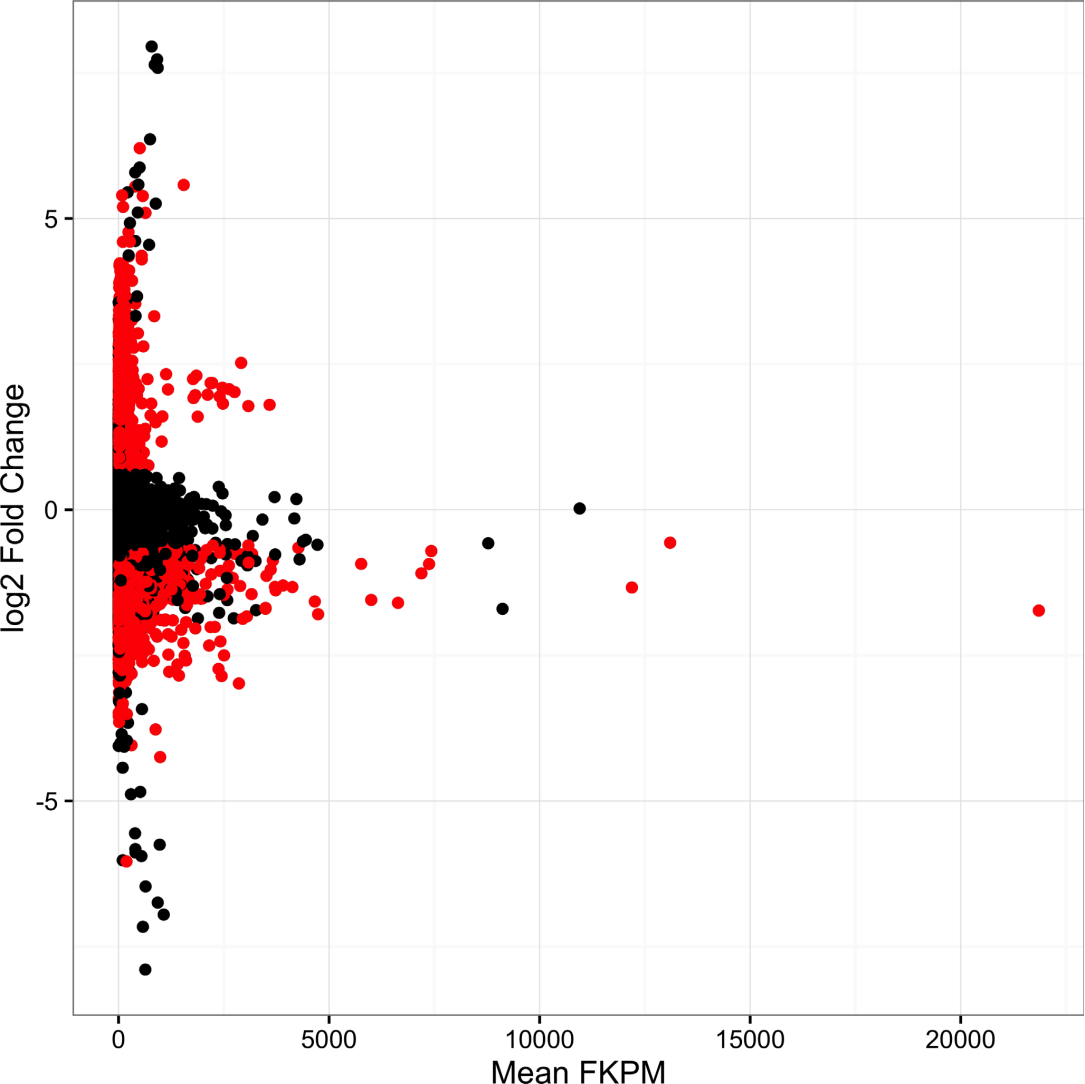
Log2 fold change versus FKPM (Fragments Per Kilobase Million) for each contig. Significantly DE contigs (FDR < 0.05) are colored in red.

Of the 37 million paired-end reads obtained approximately 41% mapped uniquely to the reference transcriptome. We found around a third of the contigs tested (11178) were significantly DE between the two female types. Of these, around half of the contigs were upregulated (5353) and half downregulated (5825) between ND and D females (Fig 1, S1 Table).

#### Cluster groups created with the GO Term enrichment analysis

Using DAVID we identified a total of 15 significantly enriched gene clusters (Enrichment score > 1.3, which corresponds to P < 0.05) between D and ND females (Table 1, significant orthologs in each of the clusters are given in Tables A-O in S1 File). Many of these clusters (1, 3, 6, 11, 12 and 14) were connected to DNA replication and are likely to be related to different oogenesis processes in D and ND females. Moreover, cluster 9 included down-regulated genes annotated with tudor domain protein features, including the *tudor* gene itself. *Tudor* genes are known to be involved in germ cell development and oogenesis, reflecting the differences in oocyte maturation between D and ND females. The rest of the 8 clusters were more interesting, and they are likely to include several genes with a direct link to diapause (see discussion). Two of these clusters were connected to metabolism, cluster 10 to glycolysis and glucose metabolism and cluster 13 to lipid and fatty acid biosynthesis, and one, cluster 5, to protein transporter activity. These clusters most probably represent metabolic changes occurring in D flies when they are preparing for the cold season. The remaining five clusters included two metal binding clusters (clusters 8 and 15), an actin binding cluster (cluster 2), a nucleotide binding cluster (cluster 4) and an oxidative stress cluster (cluster 7) (Table 1).

**Table 1:** Functional clusters of the DE genes.

Cluster No.	No. of contigs	E score	P-value	GO terms	Up/downregulated genes
1	104	6,82	1,51E-07	DNA replication, nucleic acid binding	*Nf1/DNApol-α180*
2	147	2,89	0,001	Actin binding, cytoskeletal protein binding	***Mch***, ***Act****/CG18190*
3	98	2,73	0,002	ATP-dependent helicase activity	*futch/CG11403*
4	606	2,22	0,006	Nucleotide, ATP and nucleoside binding	*Hsp70Cb*, ***cpo*, *Act/****greatwall*
5	172	2,1	0,008	Protein transporter activity	***Mhc****/CG3509*
6	14	1,89	0,013	Nuclear chromosome,	*sallimus/DNApol-α50*
7	12	1,71	0,019	Oxidoreductase activity, response to oxidatative stress	*Irc/Pxt*, *Duox*
8	22	1,71	0,019	Metal and iron-sulfur binding	*CG8102/Acon*
9	28	1,7	0,02	Maternal tudor protein	*CG17454/CG15930*
10	42	1,58	0,026	Glycolysis, glucose metabolic process	*Zw/CG4747*
11	33	1,5	0,032	Replication fork, DNA replication factor C complex	*comt/CG11403*
12	42	1,47	0,034	DNA polymerase and nucleotidyltransferase activity	*mRNA-cap/DNApol-α180*
13	34	1,43	0,037	Fatty acid and lipid biosynthesis processes	***desat1*, *2*, *Inos*, *CG9747/CG33116***
14	69	1,4	0,04	DNA packaging, chromatin and nucleosome organization	*sallimus/CG3509*
15	648	1,33	0,047	Metal and zinc ion binding, zinc finger	***desat1*,*2*, *cyp12a5***, *CytP450/Phf7*, *Pxt*

Functional clusters of the DE genes (numbers given in contigs) showing differential expression between diapausing and non-diapausing females from DAVID [53], P < 0.0001. Examples of up- and down-regulated genes are given in the last column and genes marked with bold are discussed in more details in the discussion. All significantly DE genes in each of the clusters are given in Tables A-O in S1 File.

### Tissue heterogeneity: enrichment of the ovary genes

**Fig 2:**
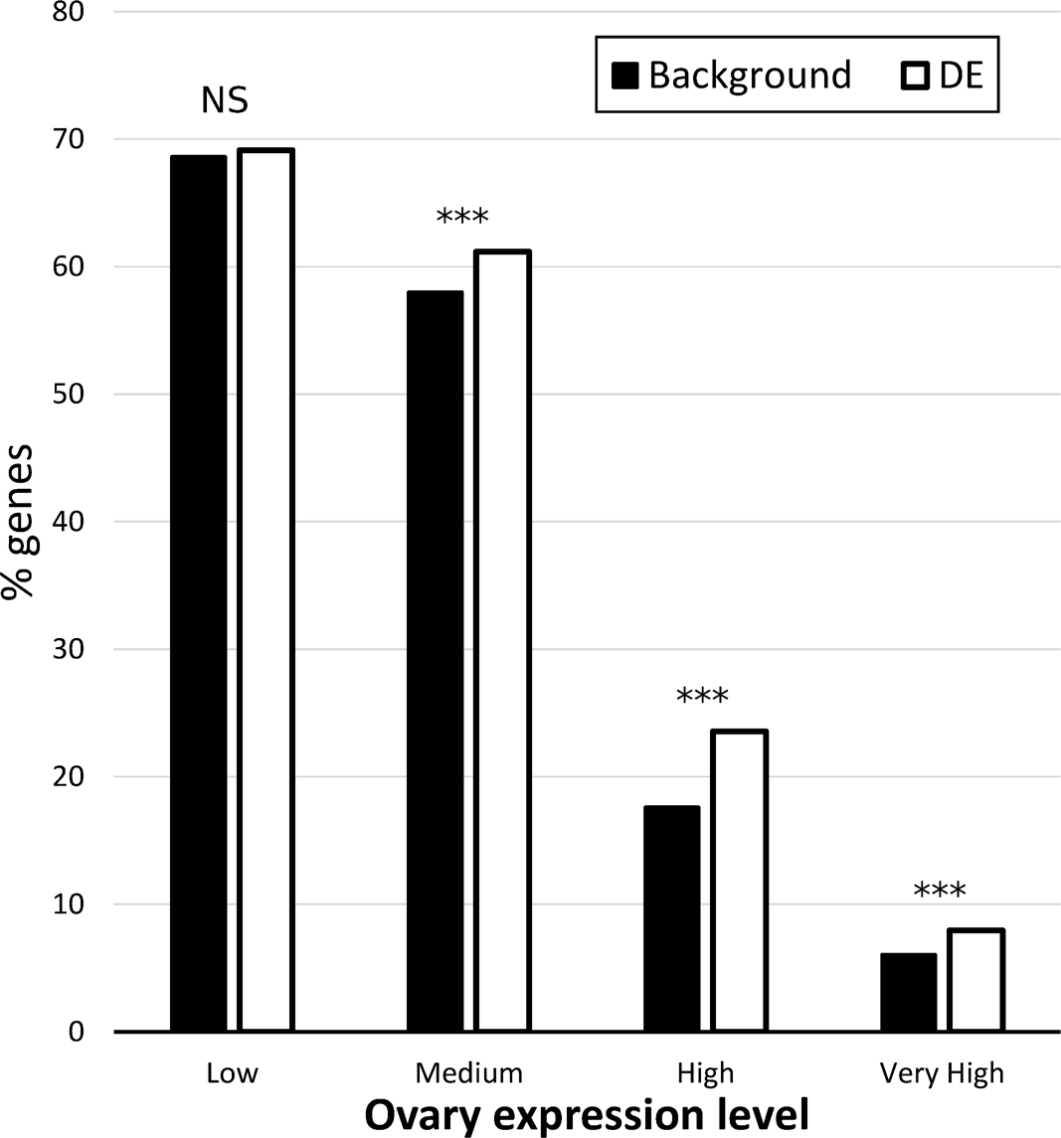
Percentage of contigs with a low, medium, high or very high expression level in female ovaries in our transcriptome data (background, black) compared to the ones found to be DE between diapausing and non-diapausing females (white). P values are from a one-sided Fisher’s exact test, see text for details: NS = P > 0.05, ***P < 0.001.

We estimated the effects of tissue heterogeneity due to ovary size on the gene expression differences between D and ND females by investigating whether DE genes were enriched for ovary expression. We found that genes categorized as having either medium, high, or very high levels of ovary expression were overrepresented in gene lists identified as DE between D and ND females (Fig 2). This finding suggests that a proportion of the genes identified as being DE were likely to due to the heterogeneous nature of the tissues compared, rather than DE.

### Validation of the RNA sequencing data and estimation of the effect of tissue heterogeneity with qPCR

**Fig 3:**
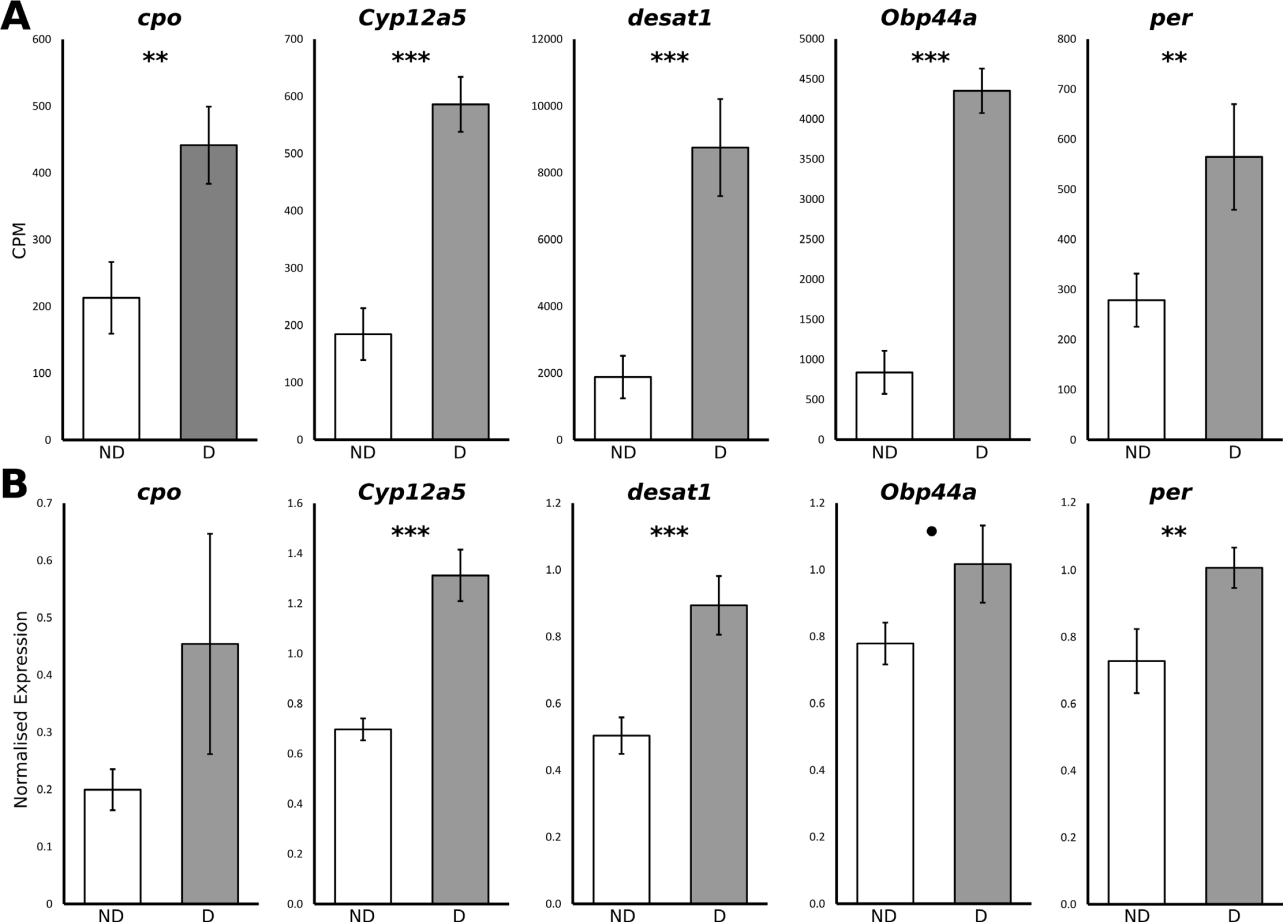
Estimating the effect of the tissue heterogenity. Normalized gene expressions levels comparing diapausing and non-diapausing *D*. *montana* females A) from the RNAseq analysis (for comparison), and B) from the qPCR using the females whose ovaries had been removed before RNA extractions. Both data sets present the combined data for all the three isofemales strains (see text for details). CPM = counts per million. Significance levels for qPCR: ^•^0.1 > P > 0.05, **0.01 > P > 0.001, ***P < 0.001.

To determine whether tissue heterogeneity between D and ND females influenced the expression patterns of the selected candidate genes, we performed qPCR runs for individual females whose ovaries had been removed. qPCR produced similar upregulation patterns as observed in the RNAseq for three out of five studied candidate genes (Fig 3), with significant expression level differences between the two female types for *cyp12a5* (P = 0.00001), *desat1* (P = 0.00026) and *per* genes (P = 0.0078) and suggestive differences in *Obp44* (P = 0.07737). The same pattern was also seen in *cpo*, but the difference remained non-significant (P = 0.15457) due to high variance between the samples (Fig 3).

### Transposable elements

We found 15 unique TE groups to be significantly DE between D and ND *D*. *montana* females. The majority of the groups (e.g. polyproteins and retrotransposons) showed upregulation and only three (e.g. *Xanthias*, *Hobo*) were downregulated in D females while different contigs annotated as *Gypsy protein* showed both up and down regulation (see S3 Table for more details).

## Discussion

Adult photoperiodic reproductive diapause is a common over-wintering strategy in temperate insect species. Here, we identified functional gene clusters and genes involved in diapause in *D*. *montana* by comparing gene expression differences between diapausing and non-diapausing females reared under the same environmental conditions. We found that around a third of the contigs in our transcriptome were DE, showing that diapause has a profound effect on female *D*. *montana* gene expression. This was somewhat expected as diapause is a complex trait, known to cause large shifts in reproductive state, cold tolerance, and behavior. Since D and ND females differed dramatically in the relative sizes of their ovaries, some of the contigs we identified as being DE were a result of comparing heterogeneous tissue types. Consequently, as we expected that genes over expressed in ovaries would be more likely to be affected by this issue, we determined whether DE contigs were enriched for genes expressed in the ovary. We found that our DE contigs were enriched for genes expressed at least at moderate levels in the ovary suggesting that some of these contigs were likely to be false positives due to the influence of tissue heterogeneity. Despite this, we note that the magnitude of the overrepresentation was relatively small (Fig 1), and in fact it could also be expected because contigs involved in reproduction would be also involved in controlling reproductive diapause. In addition, we examined changes in expression in ND and D females when their ovaries had been removed for a few key genes and found that the differences were similar to those obtained from RNAseq. Taken together, this suggests that tissue heterogeneity may be responsible for a small but significant proportion of the contigs identified as DE, and hence when evaluating the results of the present study, we focused on the gene clusters/genes with no direct connection to ovaries or/and oogenesis.

There were 15 significantly enriched gene clusters, where several genes showed differential expression patterns between D and ND females. Three of these clusters were involved with glycolysis and glucose metabolism, fatty acid and lipid biosynthesis and protein transporter activity, all of which are suggested to represent metabolic changes occurring in diapausing flies when they are preparing for the cold season. In addition, five of the clusters were connected to metal, nucleotide and actin binding and/or to oxidative stress, which may also play a role in diapause. All clusters contained new candidate genes for diapause in addition to the previously identified genes. The last seven clusters were connected to DNA replication and are thus more likely to be linked with oogenesis rather than the diapause *per se*. The lists of all genes in each of the clusters are given in Tables A-O in S1 File.

### Genes connected to metabolic changes when diapausing flies are preparing for the cold season

In the current study, DE orthologs involved with glycolysis and glucose metabolic processes are most probably linked to metabolic changes which occur when the flies begin to go into diapause. The most common cryoprotectant is glycerol, but many other polyols and sugars, such as glucose, can also function in this way [61]. Moreover, the function of glucose-6-phosphate dehydrogenase (G6PDH) coded by *Zw* in *Drosophila* affects the synthesis of cryoprotectant polyols in insect larvae [62] and this gene was found to be upregulated in D females in our study. Overexpression of *Zw* has been also associated with increased life span in *D*. *melanogaster* [63] and G6PDH is a key enzyme in NADPH synthesis which has been suggested to directly function as an antioxidant [64]. Diapausing *D*. *montana* females are known to be more cold tolerant than the non-diapausing ones, and decreases in photoperiod and/or temperature has also been found to increase cold tolerance [65]. Glucose is known to be stored in the diapausing flies mainly as glycogen. In our earlier metabolic analyses on *D*. *montana*, glucose levels of D females were found to increase almost 2-fold in autumn [65]. However, glucose may not to be the major metabolite in diapausing *D*. *montana*, as myo-inositol increased up to 144-fold during the winter in both sexes of this species [65]. We have also found that *Inos*, which encodes the enzyme myo-inositol-1-phosphate synthase, is connected to cold acclimation in *D*. *montana* and *D*. *virilis* [47]. Because *Inos* was up-regulated in D females compared to the ND ones in a constant environment, we suggest that the expression of this gene can increase during diapause without a decrease in temperature. *Inos* has also been connected to increased cold tolerance in some Coleoptera and Lepidoptera species [66,67], to egg diapause in migratory locusts [68] and to pupal diapause termination in the flesh fly *Sarcophaga crassipalpis* at the protein level [69].

*Desaturase* genes (*desat1 and desat2*) function in fatty acid metabolism and lipid metabolic processes, and were found to be upregulated in diapausing *D*. *montana* females. The finding is in accordance with our earlier microarray studies, where *desat1* and *desat2* were upregulated in *D*. *montana* females at the initiation stage, and *desat2* in the termination phase of diapause [56]. The same genes were found to be down-regulated in cold acclimated non-diapausing flies, *desat1* in *D*. *montana* and *desat2* in *D*. *virilis* [56]. *Desat2* is also known to play a role in desiccation [70] and cold and starvation tolerance [71]. Reynolds and Hand [72] showed upregulation of Δ9-desaturase during diapause in the cricket *Allonemobius socius*, and Sim and Denlinger [73] detected similar increases in *Culex pipiens*. Desaturases have also been found to play a role in pheromone biosynthesis in insects (e.g. [74]) which may explain why diapausing *D*. *montana* females are not attractive to males [75]. We also found another gene with acyl-CoA Δ11-desaturase activity, *CG9747*, to be upregulated in diapausing females. This gene has been found to show high expression levels in the initiation and early maintenance phases of *D*. *montana* diapause [59] and recently Kučerová et al. [76] have linked it to diapause in *D*. *melanogaster*. Desaturases have also been shown to increase membrane lipid unsaturation and maintenance of membrane fluidity in starvation-induced autophagy in *Drosophila* [77]. Finally, in addition to desaturases, we found that six odorant binding protein genes showed differential expression between D and ND females (S4 Table), although none of them were found to belong in any of the significantly enriched gene clusters in this study. Connection of odorant genes to diapause clearly demands future studies.

Cytochrome P450 genes form another important group of genes involved in the metabolism of steroids and fatty acids [78] and some of these genes have been connected to larval diapause in the silkmoth, *Antheraea yamamai* [79]. We found 13 *cytochrome P450* orthologs (e.g. *cyp12a5*) from the metal ion binding cluster (cluster 15, Table O in S1 File) that showed different expression levels in D and ND females, suggesting that these genes may function in *D*. *montana* diapause. Interestingly, *cytP450* genes have been associated with insecticide resistance [80,81], stress resistance in aging flies [82] and immunity (e.g. [83]). Insecticide and stress resistance, as well as immunity, may share many metabolic pathways and pleiotropic genes, but more in-depth studies are needed to clarify the connection between these traits and diapause.

*Myosin heavy chain* (*Mhc*) and several *actin* genes (from protein transport, nucleotide and actin binding clusters) were upregulated in D females in our study. Myosin is associated with muscles, where it drives muscle contraction by a cyclical interaction with actin, and both above-mentioned genes also play a role in the induction of structural changes in the cytoskeleton. Cytoskeleton structure changes at low temperatures and this has been found to increase cold tolerance in plants [84,85]. In insects, low temperatures have been found to induce changes in the distribution and structure of actin [86]. In diapausing individuals these changes can be even more pronounced than in non-diapausing ones, and are thus expected to be accompanied/caused by the upregulation of *actin* genes [86,87]. Interestingly, in *D*. *americana*, *ActinD1* expression levels have been also shown to reflect observed differences in life span of both diapausing and non-diapausing flies [88].

Molecular chaperones and cryoprotectants, like the heat shock proteins, are often synthesized to protect proteins and tissues in stressful conditions, such as freezing or desiccation [19,89], and different HSPs have been found to be active in diapausing individuals (e.g. [19,42,59,90]. However, we did not detect any significantly enriched clusters containing stress-related genes even though several heat shock protein (*Hsp*) genes were significantly DE in diapausing flies (S5 Table). Only two *Hsp* genes (*Hsp22* and *Hsp67Bc*) were significantly upregulated in diapausing *D*. *montana* females. Interestingly, Kučerová et al. [77] also found *Hsp67Bc* to be upregulated while *Hsp22* was downregulated together with other small heat shock proteins (*Hsp23*, *Hsp26* and *Hsp27*) in diapausing *D*. *melanogaster*. In our study, two *Hsps* (*Hsp23* and *Hsp26*) were downregulated in diapausing females. Furthermore, several heat shock cognate genes (e.g. *Hsc70* group genes) showed altered levels of expression in the present study (S5 Table) compared to our previous microarray study [59] where only *Hsc70-2* showed upregulation during different phases of diapause. Clearly, heat shock genes do not seem to constitute a homogeneous group of genes, but rather to show both up- and down-regulation depending on the taxonomic group and environmental conditions in the species and population home site.

### DE genes showing multiple transcripts

Expression patterns were determined for the original contigs instead of genes, because there were more than 4000 contigs that matched only to general genomic sequences in our study. It is likely that many of them are actually transcripts from a known gene, which fall outside the current gene model boundaries. In fact, further examination found that several contigs in our assembly contained regions that aligned to regions annotated as introns in other species and many of them were from genes already identified in our transcriptome. Thus, it is likely that several of our contigs represent alternative transcripts of a single gene. For example, *trehalose-6-phosphatase synthase 1* (*Tps1*), *CG1648* (molecular function not known) and *CG6426* (having lysozyme activity), showed expression differences in some contigs but not in others. Among these genes *Tps1* has earlier been connected to diapause of Onion maggots [91] and trehalose is one of the metabolites connected to the seasonal improvement of cold tolerance in *D*. *montana* [65]. On the other hand, to our knowledge *CG1648* and *CG6426* have not been connected before to diapause, but Zang et al. [92] have suggested that expression of *CG1648* is sensitive to the cold exposure in *D*. *melanogaster*. Such genes are interesting, not only as candidate genes for diapause processes *per se*, but also because they are candidates for investigating how genes in diapause may be isoform-specific.

### Transposable elements in diapause

The possible role and/or the importance of the transposable elements (TEs) in diapause is currently unknown, but several studies have connected TEs to this trait. For example, one retrotransposon has been shown to be highly expressed in the early pupal diapause of *S*. *crassipalpis* [88]. Moreover, Yokum et al. [93] showed the presence of miniature subterminal inverted repeat-like elements (MSITE) within the promoter regions and introns of diapause regulated genes *DAT-2* and *DAT-3* (diapause-associated transcripts) in *Leptinotarsa decemlineata*. These authors suggested that this may indicate a possible role of TEs in the evolution and regulation of diapause on Colorado potato beetle [93]. In our study, we found 15 unique TEs to be significantly DE between D and ND *D*. *montana* females; the majority of these TEs showed upregulation and only three of them were downregulated in diapausing females (S3 Table). Among these there were TE classes such as polyproteins, retrotransposons and non-long terminal repeats but also well characterized TEs like *Ulysses*, *Gypsy*, and *Penelope*, known from *D*. *virilis* group species [94]. Evidently, the clarification of the overall role of TEs in diapause and/or in the function of specific diapause connected genes demands more detailed research.

## Conclusions

We used RNA sequencing to study genes expression changes associated with reproductive diapause in the northern fly species *D*. *montana*. Our results demonstrated large gene expression differences between the diapause phenotype, capable of surviving the long winters, and the non-diapausing summer phenotype. This is in accordance with the view of diapause being an alternative dynamic adaptation and not just an arrest in the normal active summer development. We also found several functionally enriched gene clusters associated with metabolism, fatty acid biosynthesis and metal binding, including genes with connection to cuticular hydrocarbons (*desat* genes) or having acyl-CoA Δ11-desaturase activity (*CG9747* and several odorant binding genes). In addition, we found several transposable elements (TE) to be differentially expressed between the two female types suggesting that TEs may play a role in, or be influenced by diapause. Finally, we showed that comparing transcriptional changes in the diapausing and non-diapausing females collected from the same environmental conditions produced comparable results, even though tissue heterogeneity has to be taken into account.

RNAseq data has been submitted to NCBI's Gene Expression Omnibus. The transcriptome assembly has been deposited at DDBJ/EMBL/GenBank under the accession GECM00000000. The version described in this paper is the first version, GECM01000000. Raw reads have been deposited in GEO under accession codes: SRR2910695, SRR2910698, SRR2910701, SRR2910702, SRR2910703, and SRR2910704.

## Supporting Information

S1 File Compressed zip file of Tables A-O including significant gene clusters from DAVID analysis. Table A. Cluster 1 DNA replication. Table B. Cluster 2 Actin binding. Table C. Cluster 3 Helicase activity. Table D. Cluster 4 Nucleotide binding. Table E. Cluster 5 Protein transporter activity. Table F. Cluster 6 Nuclear chromosome. Table G. Cluster 7 Oxidative stress. Table H. Cluster 8 Metal binding. Table I. Cluster 9 Maternal tudor protein. Table J. Cluster 10 Glycolysis. Table K. Cluster 11 DNA replication. Table L. Cluster 12 DNA polymerase. Table M. Cluster 13 Fatty acid biosynthesis. Table N. Cluster 14 DNA packing. Table O. Cluster 15 Metal ion binding.
(ZIP)

S1 Table Differentially expressed contigs. (XLSX)

S2 Table Primers for quantitative PCR with the efficiency (E%) and R^2^ values. (DOCX)

S3 Table Transposable elements. (XLSX)

S4 Table Olfactory genes. (XLSX)

S5 Table Hsp genes. (XLSX)
