# Supplementary material for: Transcriptional Differences between Diapausing and Non-Diapausing D. montana Females Reared under the Same Photoperiod and Temperature
Source: PLoS One. 2016 Aug 29;11(8):e0161852. doi: 10.1371/journal.pone.0161852 (PMC5003386; doi:10.1371/journal.pone.0161852)
Supplement: S2 Table — (DOCX) [file pone.0161852.s003.docx]

Supplementary Table S1. Primers used in the qPCR analysis with efficiency (E%) and R^2^ values.

Gene Primer sequences 5' - 3' (F/R) E% R2

*cpo* GCACACTTTTCGTCAGTGGTT 101,7 0,981

GTGGTTTGGGTTTGCTCACT

*per* TTGCCGCAGGATCTCATTGGAC 93,3 0,970

TTCAGGGTCCCAAGCAGTGCAAT

*Obp44A* CTTGATTGGCCTGGCCTC 96,9 0,999

TGCGTGTGATCTCGTCGTC

*Desat 1* GCCGTCCCTATGACTTCATC 102,0 0,996

AGACGCTCTTTAGGTCATAAGC

*cyp12a5* GCGATAACAGACCAGCGTTG 104,7 0,996

CCATAGTCTCTACGCATTGCCA

*RpL32* CATCAGCAGCACCTCCAGTTC 98,4 0,998

GATATGCCAAGCTGTCGCACAA

*18S* AATGCACCGAGGAGGAGGTTGA 97,6 0,999

CGACCTGTAGTTTTGGTGTGCTGG
